# Provident Dispensaries

**Published:** 1899-10-21

**Authors:** 


### PROVIDENT DISPENSARIES.

At the recent Conference on Hospital Reform Mr. Warren, secretary of the Metropolitan Provident Medical Association, read a paper descriptive of the objects and organisation of that institution. He said the present hospital system resulted in the treatment of a million and a half of patients in the out-patient and free dispensaries every year; in other words, not counting hospital in-patients, one-third of the population of London applied every year to charity for medical relief. He was not prepared to say that any of these recipients of hospital out-patient treatment belonged to the well-to-do classes, and it might be that the lady in silks driving up in her own brougham to get free hospital treatment was more of a myth than a reality. But he thought he would be fairly stating the case when he said that a very considerable number of these people could, if they would, make provision for their ordinary doctoring, and ought only to use the out-patient departments when they required the advice of a consultant. The present habit of treating the hospitals as the family doctor was bad for them, because they were getting from charity what they were able to pay for; bad for those who really wanted the consultant advice from the hospital, because of the discomfort and delay consequent on the overcrowding of the out-patient room; bad for the hospitals, because it imposed on them a heavy financial burden; and bad for the outside practitioner, because it tended to deprive him of the means of making a decent living. And while these were the evils which had to be faced, none of the reforms as yet suggested within the out-patient department, such as the appointment of inquiry officers, the fixing of an arbitrary limit to the number of new out-patients admitted each day, or the exaction of a small payment towards the hospital expenses, could be regarded as bringing us within reach of any real solution of the difficulty. Having asked the question, "Can a solution be found in provident dispensaries?" the author proceeded to describe the spread of provident principles in application to this form of medical relief during the last 70 years, the oldest existing provident dispensary in the London district having been founded at Sydenham in 1827. The first serious attempt to deal with the medical needs of the working classes in London by means of an organised system of provident dispensaries was made in 1880, when the Metropolitan Provident Medical Association was called into existence. Mr. Warren went on to explain the objects and organisation of the association, stating that the purpose of its founders was to remedy the evils of the out-patient departments and free dispensaries by a method which should satisfy doctors as well as patients, and should be free from the abuses of the club system which flourished in many provincial towns. It had, however, been prevented from attaining any great development in London, chiefly because the free out-patient departments held the field. The objects were to be attained by the formation, in suitable districts, of provident dispensaries or medical clubs as branches of the association, under the management of local committees, with a medical staff selected from the general practitioners of the district. The association had now 20 branches in various parts of London—15 dispensaries and five medicine clubs—worked on the lines indicated in the paper. The association only allowed properly qualified medical men to join the staff, and had in the hundred or more attached to the various branches a body of professional men of whom any organisation would very rightly be proud. Each member was expected to select his own doctor from the staff and stick to him. The result was likely to be, in ordinary cases, a far more satisfactory kind of treatment than that given in the crowd and hurry of an out-patient department. The dispensaries were intended to be self-supporting. They were not actually so at present in most cases, but that was because of the severe competition against which they had to fight. Among several difficulties against which the association had to contend, Mr. Warren quoted the continued opposition of a section of the medical profession. This was much less pronounced than at the commencement of the association's work, but it still existed. The hospitals had not extended practical sympathy and support. They had done little more than profess for the provident dispensary system a theoretical admiration that had refused to recognise that their out-patient departments under present conditions were rivals with which it was almost hopeless for provident dispensaries to contend. With one exception the most successful provident dispensaries were those farthest from hospitals. The Royal Free Hospital had set an example to other hospitals in the matter of practical co-operation with provident dispensaries. The writer proceeded to detail what was being done by way of an experiment between the Metropolitan Provident Medical Association and the Hospital Saturday Fund in the direction of keeping unsuitable cases out of the hospitals. From 30,000 to 40,000 persons were enrolled as provident members of the various branches of the association, and they contributed upwards of £5,500 towards their medical treatment, £4,649 of which was devoted to the payment of the medical men, including drugs and expenses of dispensing. He thought it was not unreasonable to assume that but for these provident dispensaries only a small proportion of this amount would find its way into the pockets of the general medical practitioner. The provident dispensary movement might not for many years do all that its promoters and friends expected, but he believed it had a great future before it.

